# Awake Extracorporeal Membrane Oxygenation for Acute Respiratory Distress Syndrome: Which Clinical Issues Should Be Taken Into Consideration

**DOI:** 10.3389/fmed.2021.682526

**Published:** 2021-07-01

**Authors:** Xin Yu, Sichao Gu, Min Li, Qingyuan Zhan

**Affiliations:** Department of Pulmonary and Critical Care Medicine, Center of Respiratory Medicine, National Clinical Research Center for Respiratory Diseases, Institute of Respiratory Medicine, China-Japan Friendship Hospital, Beijing, China

**Keywords:** extracorporeal membrane oxygenation, acute respiratory distress syndrome, mechanical ventilation-induced lung injury, spontaneous breath, respiratory drive

## Abstract

With the goal of protecting injured lungs and extrapulmonary organs, venovenous extracorporeal membrane oxygenation (VV-ECMO) has been increasingly adopted as a rescue therapy for patients with severe acute respiratory distress syndrome (ARDS) when conventional mechanical ventilation failed to provide effective oxygenation and decarbonation. In recent years, it has become a promising approach to respiratory support for awake, non-intubated, spontaneously breathing patients with respiratory failure, referred to as awake ECMO, to avoid possible detrimental effects associated with intubation, mechanical ventilation, and the adjunctive therapies. However, several complex clinical issues should be taken into consideration when initiating and implementing awake ECMO, such as selecting potential patients who appeared to benefit most; techniques to facilitating cannulation and maintain stable ECMO blood flow; approaches to manage pain, agitation, and delirium; and approaches to monitor and modulate respiratory drive. It is worth mentioning that there had also been some inherent disadvantages and limitations of awake ECMO compared to the conventional combination of ECMO and invasive mechanical ventilation. Here, we review the use of ECMO in awake, spontaneously breathing patients with severe ARDS, highlighting the issues involving bedside clinical practice, detailing some of the technical aspects, and summarizing the initial clinical experience gained over the past years.

## Background

For years, invasive mechanical ventilation has been the first-line tool for managing severe acute respiratory distress syndrome (ARDS). However, ARDS patients treated with conventional mechanical ventilation are at high risk of detrimental complications, including ventilator-associated pneumonia (VAP) (1), mechanical ventilation-induced lung injury (VILI) (2), and diaphragm atrophy or myotrauma (3). To avoid iatrogenic injuries to the lungs and extrapulmonary organs associated with intubation and invasive mechanical ventilation, as well as subsequent side effects related to adjunctive therapies of sedatives, opioids, and neuromuscular blocking agents, venovenous extracorporeal membrane oxygenation (VV-ECMO) has gradually come to be a preferable treatment for refractory respiratory failure (4, 5). Some centers have even pursued the idea of using ECMO as a first-line treatment to rest the heart and lungs, to facilitate protective and even ultraprotective ventilation with low tidal volume, low frequency, low platform pressure, low driving pressure, and low mechanical power (6–10).

In recent years, the new concept of “awake ECMO” has emerged, with ECMO being used for awake, non-intubated, spontaneously breathing patients with respiratory and circulatory failure (11). Although this new technique seems promising as an alternative to mechanical ventilation (12–14), high-quality evidence regarding its safety, feasibility, and efficacy remains sparse. In the present review, we aimed to discuss the pivotal issues and share our initial experience in using VV-ECMO in awake, spontaneously breathing patients with severe ARDS.

## Potential Indications of Awake ECMO

The first attempt at awake ECMO was reported in a population with an end-stage pulmonary disease as an approach to bridging to transplant (15, 16). With the support of ECMO alone, this group of patients could reserve spontaneous breath and free from symptoms of dyspnea, facilitating early ambulation and rehabilitation, thus being rationale to improve both short- and long-term outcomes (16, 17). Recently, several case series with small sample size reported the use of ECMO in non-intubated, primarily ARDS patients, of which the etiology of ARDS includes perioperative lung injury, multiple trauma, viral pneumonia of influenza and COVID-19, and *Pneumocystis jirovecii* pneumonia (12, 13, 18–21). We reviewed these cases' characteristics and summarized several patient selection criteria combined with our center's experience.

### General Criteria

As a complex and resource-consuming intervention, a careful weighing of the potential benefits and risks of ECMO by using predictive survival models and adequate communications between doctors and patients/surrogates before its initiation are crucial (22). As a prerequisite, patient selection should first meet the criteria for conventional ECMO, which, based on the severity of hypoxemia, respiratory mechanics and radiological features as reflected in the Murray score (23), and the patients' outcomes, were usually evaluated by the prognostic scores using PRESERVE, RESP, or PRESET score (24–26). Though any evidence-based guidelines or expert consensus has not recommended it, awake ECMO seems more inclined to benefit ARDS patients at the early phase of the disease (27, 28), as it appears to be safer and more feasible in those with a better level of oxygenation, less accumulation of airway secretions, a smaller area of consolidation, and fewer accompanied dysfunctional organs (29).

### Immunosuppressed Patients

As reflected in predictive survival models of ARDS (25, 30–32), immunosuppression independently predicts worse outcomes, with even higher mortality in patients supported by ECMO. A recent retrospective study involving a total of 288 severe ARDS patients requiring ECMO support showed that immunosuppressed ones had both lower survival rates and ventilator-free days (33) despite the diverse survival rate among the different etiologies of immunosuppression. However, it is interesting that immunocompromised ARDS patients are more likely to be selected for awake ECMO (13, 18), and the possible reasons might be as follows: (1) The immunocompromised state in these patients mainly results from HIV infection, hematological malignancies, the transplantation of solid organs, and autoimmune diseases treated with corticoids and/or immunosuppressive therapy (13, 18, 32). These patients are at high risks of opportunistic infections, usually with pathogens such as *P. jirovecii* and cytomegalovirus, which could lead to moderate or severe respiratory failure but less possibly accompanying sepsis, shock, acute kidney injury, or other extrapulmonary organ disorders (34, 35). Moreover, it would be relatively safer and more feasible to adopt awake ECMO in the progressive respiratory failure induced by *P. jirovecii* and cytomegalovirus infections, which are often characterized by bilateral lung diffuse ground-glass lesions without patchy consolidations or much airway secretions. (2) Previous evidence concerning ARDS patients supported by ECMO mainly was obtained from intubated ones, among whom the mortality was mainly associated with VILI and nosocomial infections (36, 37). Of note, poor outcomes were rarely found resulted from the mechanical complication of ECMO (36, 37). Thus, it might be possible that avoiding intubation and invasive mechanical ventilation might substantially improve the outcome of ARDS patients.

## Etiology of ARDS

The etiology of ARDS and the factors driving acute deterioration of respiratory dysfunction are crucial when choosing the candidate for awake ECMO. Usually, lung function concerning gas exchange can be restored in days or weeks after the alleviation of driving factors in the mild and moderate ARDS population. Under given circumstances, the direct insult of driving factor and the secondary histopathologic changes of lung injury, including diffuse alveolar damage, hemorrhage, and fibrosis, however, would take a prolonged period to recover and even become irreversible. In some patients, hypoxemia and hypercapnia might persist and even progress for the increase of alveolar dead space and intrapulmonary shunt despite effective control of pathogenic stimuli, especially in patients with existing lung disease. One study has shown that pulmonary disorders such as pneumonia, aspiration, or lung contusion might be more prone to induce early lung fibrosis as compared with extrapulmonary disorders (38), which could result in an irreversible progression of the lung lesions, eventually leading to the failure of weaning from ECMO. This group of patients would have no choice but to bridge to lung transplant. Unfortunately, evidence regarding the underlying mechanism of disease exacerbation and how to predict the reversibility of rapid progression of disease remains scarce (32, 39, 40). Moreover, it is not rare that infections or other pathogenic insults could exacerbate previous pulmonary lesions. Among immunosuppressed ARDS patients, those with anti-synthetase syndrome, rheumatoid arthritis, and Sjogren's syndrome, compared with patients with HIV or solid organ transplantation, were found to be less likely to benefit from ECMO support, perhaps due to a higher probability of existing interstitial lung disease (ILD) in this population and opportunistic infections might drive a rapid but irreversible progression of ILD (32, 41–43). In brief, a careful selection of patients who are suitable for awake ECMO is a crucial issue and high-quality evidence is needed.

## Monitoring and Modulation of Respiratory Drive

For ARDS patients, the adverse effects of either over strong or weak spontaneous breathing have been widely discussed in the aspect of respiratory drive, the course of the disease, and the severity of lung injury (44–47). Excessive spontaneous breathing, as confirmed in many studies, may lead to “patient self-inflicted lung injury (P-SILI),” especially in ARDS patients with “baby lungs” (2, 48). An appropriate level of spontaneous breathing could help improve oxygenation, optimize ventilation–perfusion matching (49), and avoid diaphragm dysfunction (50). One of the most important goals of the VV-ECMO was to protect the lungs from further injuries by reducing tidal volume, respiratory rate, plateau pressure, driving pressure, and mechanical power.

### Control of Respiratory Drive

In the physiological state, the respiratory drive is mainly regulated by the cerebral cortex, metabolic feedback, and chemical feedback, among which chemical feedback of blood PaCO_2_/pH level plays a dominant role (51). In some cases, however, dyspnea symptoms are not significantly alleviated, even when effective oxygenation and decarbonation are attained with the support of VV-ECMO (52). Crotti et al. reported that only 27% (8/30) of ARDS patients could tolerate awake VV-ECMO in lieu of mechanical ventilation, and 50% of these patients maintained an unexpectedly high respiratory rate even with an increased ECMO sweep gas flow rate as high as 12–15 L/min (29). In severe ARDS patients, the development of an excessively strong respiratory drive might be caused by atelectasis, microthrombosis, or inflammation-induced activation of physiological receptors in the lungs or thorax (53). Other explanations for respiratory control disorders include a separation of the “brain curve” (respiratory drive) and the “ventilation curve” (actual ventilation) and an upwards shift in the metabolic curve (metabolic hyperbola) caused by an elevation in dead space after high-frequency ventilation (54).

### Monitoring of Respiratory Drive

Respiratory drive monitoring and modulation are one of the key issues in determining the feasibility of awake ECMO in severe ARDS patients. The intensity and amplitude of the respiratory drive can be accurately evaluated in patients receiving invasive mechanical ventilation by collecting indicators such as respiratory rate, tidal volume, minute ventilation, inspiratory flow rate, the electrical activity of diaphragmatic muscle (EA_di_), esophageal and gastric pressures, and airway obstructive pressure (P_0.1_). Although there had been fewer monitoring options for awake non-intubated patients than in those receiving invasive mechanical ventilation, it was not difficult to recognize high respiratory efforts by the presence of clinical signs of dyspnea and respiratory distress, a rapid shallow breathing pattern and signs of agitation. Besides, among patients receiving non-invasive mechanical ventilation (NPPV), more objective and quantitative parameters like respiratory rate, minute ventilation, waveforms of dys-synchrony, and P_0.1_ could be monitored. Moreover, with the widespread use of bedside ultrasound, diaphragmatic excursion, and diaphragm thickening fraction can be good parameters to evaluate inspiratory effort in both intubated and non-intubated patients. As there is growing interest in the role of accessory muscles in critical illness, ultrasound might also be of great value in assessing the structure and activity of accessory muscles (55).

## Maintaining ECMO Blood Flow

### Configuration of Cannulation

In most clinical practice, the blood flow of VV-ECMO is generally recommended to establish with two-cannula access-drainage from the femoral vein and reinfusion into the internal jugular vein (56). An ECMO blood flow obtained with a 21–23 Fr venous drainage cannula could reach as much as 60% of the cardiac output in ARDS patients (57). So far, there is no known difference in the drainage efficiency between multistage drainage cannula with side holes and most distal tip drainage cannula (58); however, in some situations with the relative inadequacy of intravascular volume, stable ECMO blood flow might not be easily maintained through a multistage cannula due to the collapse and variability of the inferior vena cava (IVC) if the top of draining cannula is located far away from the right atrium. On the other hand, there could be a potential risk of an unacceptable degree of recirculation as the tops of drainage and inflow cannulas are positioned too close, which could compromise the oxygenating efficiency of ECMO. Thus, alternative single-site access involving the internal jugular vein with a double-lumen cannula (mostly 27–31 Fr) could be recommended in patients receiving awake ECMO because the large lumen provides full oxygenation support through enough drainage and minimizing recirculation if proper positioning and monitoring could be achieved (59, 60). Moreover, awake VV-ECMO with double-lumen cannula enables early rehabilitation and improves outcomes (61).

### Heart–Lung Interaction

In VV-ECMO, classic venous drainage is achieved through femoral vein access, with the top advanced at the junction between the IVC and the right atrium. Compared with the ones under spontaneous breathing, a larger end-expiratory lung volume could remain among patients under positive pressure ventilation, resulting in a relatively caudal position of the diaphragm and a consequent better venous drainage (62). For patients undergoing awake VV-ECMO, the tidal volume and respiratory rate may fall quickly following cannulation and extracorporeal support initiation, resulting in a low residual capacity and even atelectasis. As a consequence of heart–lung interactions (63), the position of the drainage cannula tip moves further away from the right atrium as the diaphragm moves toward the cranial side. Significant IVC collapse, induced by a dramatic shift in intrathoracic pressure during spontaneous breathing, especially in the inspiratory phase (11) or under a relatively conservative fluid management strategy (64, 65), might cause unstable blood drainage of ECMO. Not only does the decreased ECMO blood flow itself aggravate the patient's hypoxia, but the subsequent dyspnea symptoms, in turn, affect the drainage of the inferior cannula. This vicious cycle may eventually lead to the failure of awake VV-ECMO. Therefore, stable ECMO flow relies on both proper cannulation and consideration of the dynamic swings in intravenous volume related to heart–lung interactions.

## Combined Ventilation

The periodic opening and closing of alveoli in the physiological state enables gas exchange and participates in the regulation and transportation of lung water (66, 67). Under some pathological conditions, it even helps repair and regenerate lung tissue to a certain degree. Although the function of oxygenation and decarbonation of the native lung can be replaced by ECMO, primary and heterogeneous lesions in the lung tissue, aggregation of lung consolidation, and inflammation after ECMO cannulation may hamper the repair and regeneration of lung tissue (68, 69). In addition, an increase in pulmonary vascular resistance caused by the decrease of lung volume might compromise, or even offset, the benefit of ECMO concerning reversing hypoxemic pulmonary vascular constriction (70) and thus might further compromise the protective effect of VV-ECMO against right ventricular failure (5). So, it raises a question on how to find the balance between lung rest and proper ventilatory load.

Of note, a moderate level of end-expiratory positive pressure (PEEP), ranging from 10 to 20 cmH_2_O, is essential in ultraprotective or near-apneic mechanical ventilation to keep the alveoli open and reduce shear damage (6–9, 71). Thus, the proper timing of initiating spontaneous breathing in ARDS patients and the options for combined respiratory support warrant careful consideration (44). ECMO centers favor different combined ventilation options according to their own clinical experience, including initiating awake ECMO without establishing any artificial airway, removing the artificial airway within 24–48 h after ECMO cannulation, or bridging with a tracheotomy and then weaning from positive pressure ventilation (12, 13, 72). So far, there has not been enough evidence to compare the advantages of these options regarding the safety, feasibility, protocols, and effects on survival or VAP/VILI incidence. There has been an increasing agreement on the early weaning of invasive mechanical ventilation during ECMO when doctors could confirm the efficiency of ECMO therapy, optimal hematocrit level, and no hemodynamic instability, neurological deficit, or other catastrophic complications (73). Recently, one study showed that patients taken off mechanical ventilation during support of ECMO had a higher likelihood of survival to discharge and were mobilized in half as many days (74). So far, there has been a paucity of evidence on other alternative modes of respiratory support during awake ECMO, such as high-flow nasal cannula oxygen therapy (HFNC), NPPV, or complete separation from oxygen therapy. With a comprehensive evaluation of the etiology and course of ARDS, the level of airway secretion, and the risks of VILI, we prefer HFNC or NPPV as the combined mode of ventilation in the patients at an early phase of ARDS, especially among the ones where an initiation of ECMO could dramatically decrease the level of respiratory drive. If patients present progressive dyspnea, excessive respiratory secretions, or concomitant organ failure during awake ECMO, early intubation should be considered.

## Sedation and Analgesia

It has been challenging to manage analgesia and sedation in both initiating and running phase of awake ECMO. Local anesthesia and general analgesia and sedation are indispensable to ensure successful cannulation, especially in ARDS patients with dyspneic symptoms due to severe hypoxia. Adequate depths of analgesia and sedation protect patients from pain and anxiety resulting from invasive procedures and discomfort of disease, and promote the level of oxygenation by providing a stable blood flow and reducing systemic oxygen consumption. A proper level of analgesia and sedation also enables patients to preserve proper levels of spontaneous breath and early ambulation. However, an overdose of sedative and analgesic agents may lead to a deteriorating respiratory and circulatory failure and even result in intubation and invasive mechanical ventilation.

Most of the agents used for analgesia and sedation exhibit pharmacokinetic and pharmacodynamic changes in patients on ECMO as a result of both patient- and circuit-related factors (75). On the one hand, as the circuit is considered a separate pharmacokinetic compartment, it may influence drug absorption and sequestration, especially for those lipophilic and highly protein-bound ones, including fentanyl, benzodiazepines, and propofol, which could lead to an underdosing in the initial drug administration period. On the other hand, ARDS patients often present with acute kidney injury, augmented cardiac output, and increased blood volume during ECMO support, leading to changes in analgesia and sedation requirements (76).

Parenteral opioids such as fentanyl and morphine/hydromorphone have often been chosen as the preferred agents in ECMO, and hydromorphone showed less sequestration in ECMO circuits and more days alive without delirium (77). Compared with propofol or benzodiazepines, dexmedetomidine produces sedative effects without amnesia or respiratory depression, making it an attractive option for general sedation in ARDS patients undergoing awake ECMO (78). Other adjunct agents like atypical antipsychotics have also been used in ARDS patients, but the safety and efficacy in awake ECMO need to be proven. In conclusion, finding a balance between drug concentration and the goal of analgesia and sedation is essential during awake ECMO.

## Limitations

It is important to note that although awake ECMO has its specific advantages compared to conventional ECMO, there have been some potential risks and limitations. First, for a moderate to severe ARDS patient with hypoxemia under the support of HFNC or NPPV, it is more difficult to perform cannulation under the situation of dyspnea, anxiety, and agitation. On the other hand, studies have reported a higher risk of fatal mechanical complications such as decannulation in patients receiving awake ECMO, which are mostly unpredictable and unpreventable but may contribute to a lower survival rate (79). In addition, patients who present with a typical ARDS radiological feature, especially with a substantial amount of consolidation in the gravity-dependent areas of lungs, are more likely to benefit from the prone position strategy (80, 81), whereas keeping awake patients with ECMO in a prone position for a more extended period of time is much more difficult in clinical practice. Finally, concerning the assessment of the native organ's function, we would be lacking some respiratory physiological or respiratory mechanics indicators in awake ECMO patients and would rely more on clinical symptoms and signs and radiological manifestations. Considering the risks of transportation with ECMO, bedside techniques such as portable X-rays and ultrasound seem more accessible.

## Conclusions

In summary, the application of awake ECMO in severe ARDS patients seems promising as it allows several inherent side effects related to conventional mechanical ventilation to be avoided. However, it remains complicated since limited clinical data have been provided so far. By applying strict patient selection criteria, maintaining a stable ECMO blood flow during awake ECMO implementation, closely monitoring interactions between the artificial organ and the patient's native organ, and avoiding underlying fatal complications, awake ECMO could be a strong rationale to achieve organ protection and rest in ARDS patients in the future.

## Author Contributions

XY, SG, and ML have designed, written, and reviewed this paper. All authors contributed to the article and approved the submitted version.

## Conflict of Interest

The authors declare that the research was conducted in the absence of any commercial or financial relationships that could be construed as a potential conflict of interest.
